# Music Lessons Improve Auditory Perceptual and Cognitive Performance in Deaf Children

**DOI:** 10.3389/fnhum.2014.00488

**Published:** 2014-07-01

**Authors:** Françoise Rochette, Aline Moussard, Emmanuel Bigand

**Affiliations:** ^1^Laboratoire d’Etude de l’Apprentissage et du Développement (LEAD – CNRS 5022), Université de Bourgogne, Dijon, France; ^2^Rotman Research Institute, Baycrest, University of Toronto, Toronto, ON, Canada

**Keywords:** congenitally deaf children, music training, auditory working memory, phonetic discrimination, auditory perception

## Abstract

Despite advanced technologies in auditory rehabilitation of profound deafness, deaf children often exhibit delayed cognitive and linguistic development and auditory training remains a crucial element of their education. In the present cross-sectional study, we assess whether music would be a relevant tool for deaf children rehabilitation. In normal-hearing children, music lessons have been shown to improve cognitive and linguistic-related abilities, such as phonetic discrimination and reading. We compared auditory perception, auditory cognition, and phonetic discrimination between 14 profoundly deaf children who completed weekly music lessons for a period of 1.5–4 years and 14 deaf children who did not receive musical instruction. Children were assessed on perceptual and cognitive auditory tasks using environmental sounds: discrimination, identification, auditory scene analysis, auditory working memory. Transfer to the linguistic domain was tested with a phonetic discrimination task. Musically trained children showed better performance in auditory scene analysis, auditory working memory and phonetic discrimination tasks, and multiple regressions showed that success on these tasks was at least partly driven by music lessons. We propose that musical education contributes to development of general processes such as auditory attention and perception, which, in turn, facilitate auditory-related cognitive and linguistic processes.

## Introduction

One of every 800 children in France is born with congenital deafness (Chen et al., 2007). The technological development of devices for the restoration of the auditory function is progressing. Hearing aids optimize residual auditory capacities, especially at low and medium frequencies, though they remain relatively ineffective for the perception of higher frequencies. Cochlear implants are sensory aids that convert auditory information into electrical impulses transmitted to the auditory nerve through multiple stimulating electrodes (currently between 16 and 22) located throughout the cochlea. Despite low spectral resolution, reduced temporal fine-grained structure, and reduced dynamic range due to the number of electrodes (Zeng et al., 2008 for a review), those receiving pediatric cochlear implants develop higher speech and language outcomes than non-implanted peers. Hereafter in this text, the term “deaf children” will refer to both hearing aid and cochlear implant pediatric users suffering from pre-lingually profound deafness (i.e., without auditory knowledge prior to the use of their devices).

Sensory deprivation has long-lasting repercussions on brain development and behavioral outcomes (Pisoni et al., 2008; Kral, 2013). In deaf children, the duration of deafness (i.e., from birth to auditory rehabilitation) is negatively correlated with neural development (Kral, 2013), as well as perceptual, linguistic, and cognitive abilities (Geers et al., 2007; Pisoni et al., 2008; Peterson et al., 2010; Havy et al., 2013). As the development and organization of cortical auditory pathways critically depends on sensory experience (Kral et al., 2000; Sharma et al., 2002; Kral and Eggermont, 2007), the restoration of auditory function with technical devices is alone insufficient for the children to “hear” properly. Deaf children must learn to interpret auditory signals to build meaningful sound representations and listening strategies. This learning often has to be supported by auditory training therapies (Wu et al., 2007). In a previous study (Rochette and Bigand, 2009), we trained a small sample of severe to profoundly deaf children through interactive auditory games targeting four main auditory-related processes such as discrimination and identification of sounds, auditory scene analysis, and auditory working memory. After 20 half-hour weekly training sessions, these children showed a significant improvement in each of the trained tasks, as well as transfer of benefits to linguistic sound perception (phonetic discrimination), which had not been trained.

Music may constitute a powerful stimulus to train perceptive and auditory-related skills in deaf children. Musical activity involves a broad brain network and engages various perceptual and cognitive processes. Music practice produces neuroanatomical and neurofunctional modifications in expert musicians, but also after short periods of practice in adults and children (see Wan and Schlaug, 2010 for a review). Trainor et al. (2003) tested 4–5 years-old normal-hearing children in an EEG paradigm after 1 year of musical training with the Suzuki method to assess changes in activation patterns in response to auditory stimuli (event related potentials, ERPs). After training, children showed faster development of the auditory brain responses (enhanced early ERP components P1, N1, and P2) compared to non-musically trained matched children (see also Hyde et al., 2009 for a similar study using fMRI). Interestingly, music practice also enhances functions that are seemingly unrelated to the musical activity (e.g., Moreno, 2009). In normal-hearing children, positive effects of musical training have been observed in non-musical abilities including visuo-spatial skills (Bilhartz et al., 1999), IQ (Schellenberg, 2004; Moreno et al., 2011), phonetic discrimination (Anvari et al., 2002; Degé and Schwarzer, 2011; Chobert et al., 2012), reading abilities (Anvari et al., 2002; Moreno et al., 2009), and verbal memory (Nutley et al., 2014). In particular, the link between music and language abilities and their overlapping processes is of great interest. Music and language have common characteristics: both systems are composed of discrete elements (phonemes and notes), organized into temporal and hierarchical structures (words and chords), rely on auditory processing of complex acoustical elements and convey rich meanings (Patel, 2008). Studies have shown large overlap in brain regions involved in the processing of music and language at the cortical (Tillmann et al., 2006; Koelsch et al., 2009) and subcortical (Strait and Kraus, 2014) levels. These commonalities, in both processes and brain networks, may underlie transfer effects from one domain to another in the normal population. Thus, training with one type of material (e.g., music) should improve their efficiency to process other types of stimuli (e.g., language; see Besson et al., 2011; Kühnis et al., 2013).

To date, only a few studies have examined the effects of musical training in hearing impaired populations. After training adults for 6 months, Petersen et al. (2012) showed improvement in perception of musical acoustic features, such as timbre, melodic contour, and rhythm, as well as in perception of emotional prosody. In children, Chen et al. (2010) compared pitch interval recognition in 27 children with cochlear implants (mean age = 6.7 years). Thirteen of these children were musically trained. The perception of musical sounds was significantly better in musically trained children and correlated with the duration of musical training. This suggests that training induced experience-dependent changes in the auditory pathway. Moreover, if musical training improves general auditory perception, then it is likely that the perception of non-music sounds, such as linguistic stimuli, would also improve. Only two published studies have investigated the effect of music training on transfer to the linguistic domain in children (Yucel et al., 2009; Torppa et al., 2014). In the first (Yucel et al., 2009), 18 cochlear-implanted children were enrolled in a training program based on auditory–verbal learning. In addition to this program, nine children received musical stimulation at home from their parents. Children were tested at 1, 3, 6, 9, 12, and 24 months on phonetic discrimination, word identification, comprehension of simple auditory instructions, and sentence repetition. The music group showed greater improvement at 3 months for a task requiring the comprehension of auditory instructions, suggesting a limited effect of music training on children’s auditory perception and cognition. However, several methodological issues may have attenuated possible gains from musical training, including potential differences in the group demographics, ceiling effects in the tasks, and limitations due to parent-administered training. Moreover, the musical training was limited to pitch or rhythm discrimination in one- or two-note items. In the second such study (Torppa et al., 2014), musically trained deaf children showed improved perception of prosodic cues in words, as well as improved working memory (digit span). However, the type of music training (instrumental practice, singing, or dance) and its duration/frequency was heterogeneous and the sample of musically trained children was small (*N* = 8). Taken together, these data suggest that there is a clear need for additional studies to examine the effects of musical training on deaf children, particularly studies that address transfer to non-music abilities.

The goal of the present study was to assess whether music lessons, given in a small group setting and led by a professional music teacher, affect deaf children’s abilities in auditory perception, auditory cognition, and linguistic domains. In a cross-sectional design, we compared 14 profoundly deaf children who completed weekly music lessons for a period of 1.5–4 years and 14 deaf children who did not receive music lessons. Auditory perception (discrimination and identification tasks) was tested with environmental sounds, as well as linguistic sounds (phonetic discrimination task). Higher level of auditory processing (auditory scene analysis) and auditory working memory were also assessed using environmental sounds. As music is a rich acoustic stimulus and because of shared general mechanisms for auditory perception across domains, we hypothesized that music training would improve auditory-related performance in non-music domains, specifically an improvement in perceptual tasks using environmental and linguistic sounds, as well as in auditory scene analysis. As a result of better perception and because music training involves many cognitive processes including auditory attention and working memory, we also expected enhanced performance in higher level of auditory-related cognitive abilities such as auditory working memory.

## Materials and Methods

### Participants

Twenty-eight profoundly deaf children were recruited through the CEOP institute (Centre Experimental Orthophonique et Pédagogique de Paris, a specialized institute, which offers adapted schooling and therapies for children with severe and profound hearing impairment). Fourteen children (mean age = 8.6 years, SD = 1), enrolled full-time at the institute, followed weekly 1-h music lessons for 2.6 years on average (i.e., since their admission to the institute; SD = 0.80). The other 14 participants (mean age = 7.9 years, SD = 1.4) did not receive musical instruction. They were enrolled in the institute at 50% and followed a classic schooling program (mainstream) during the other 50%. All 28 children generally received significant auditory stimulation from their parents and from the school as they were orally educated (i.e., teaching based on auditory strategies). Children were classified as profoundly deaf (highest degree of deafness), with an unaided bilateral hearing loss of >91 dB. They were either using hearing aids alone or hearing aids in conjunction with cochlear implants^1^. Given the difficulty of recruiting a large and homogeneous sample of deaf children, we chose to mix children with these different types of auditory device, making sure that the type of device was equally represented in both groups.

*T*-test for independent samples did not revealed significant differences between groups for age [*t*(26) = 1.46, *p* = 0.16], age at correction [*t*(26) = 0.73, *p* = 0.47], duration of utilization of the device [*t*(26) = 0.75, *p* = 0.46], and type of correction [hearing aids versus cochlear implants, *t*(26) = −0.36, *p* = 0.71]. Perfect matching between experimental and control groups is extremely difficult when working with profoundly deaf children. Although the experimental group was slightly (but not significantly) older than the control group, we prioritized matching children in terms of age of hearing correction and duration of utilization of the device (i.e., length of auditory experience), and we limited our population to orally educated children only, these factors being strongly associated with the outcome of auditory abilities and language development in deaf children.

As perception of vowels relies on the perceptive analysis of formants frequencies from 2000 Hz, pure-tone detection at 50 dB (with hearing devices) was assessed for 2000, 4000, and 8000 Hz. Table 1 presents the participant details (sex, chronological age, age at correction, duration of hearing device usage, perceptual thresholds, and the type of hearing device).

**Table 1:** **Demographic data of participants (HA, hearing aid; CI, cochlear implant)**.

Participants	Sex	Age in months (years)	Age at correction in months	Duration of use of the device in months	Threshold 2000 Hz <50 dB	Threshold 4000 Hz <50 dB	Threshold 8000 Hz <50 dB	Type of device	Brand and processor of CI
**MUSIC GROUP**									
S1	M	114 (9.5)	60	54	Yes	No	No	HA	
S2	F	113 (9.4)	59	54	Yes	No	No	HA	
S3	F	119 (9.9)	18	101	Yes	Yes	Yes	CI + HA	Nucleus CP 810
S4	M	111 (9.3)	10	101	Yes	Yes	No	HA	
S5	M	104 (8.7)	39	65	Yes	No	No	HA	
S6	F	112 (9.3)	18	94	No	No	No	HA	
S7	M	97 (8.1)	10	87	Yes	Yes	Yes	CI + HA	Nucleus freedom
S8	F	84 (7)	18	66	Yes	Yes	Yes	CI + HA	Nucleus CP 810
S9	F	99 (8.3)	24	75	Yes	Yes	Yes	CI + HA	Nucleus CP 810
S10	M	108 (9)	4	104	Yes	No	No	HA	
S11	F	95 (7.9)	14	81	Yes	Yes	Yes	CI + HA	Bionics harmony
S12	F	87 (7.3)	16	71	Yes	Yes	Yes	CI + HA	Nucleus freedom
S13	F	84 (7)	7	77	Yes	No	No	HA	
S14	M	112 (9.3)	24	88	Yes	No	No	HA	

Mean		102.8 (8.6)	22.93	79.86					
SD		11.8 (1.0)	17.72	16.77					
**CONTROL GROUP**									
S15	M	88 (7.3)	28	60	Yes	Yes	No	HA	
S16	M	112 (9.3)	18	94	Yes	Yes	Yes	CI + HA	Nucleus freedom
S17	F	83 (6.9)	22	61	Yes	Yes	No	HA	
S18	F	75 (6.3)	41	4	Yes	Yes	Yes	CI + HA	Medel Sonata
S19	F	95 (7.9)	17	78	Yes	Yes	No	HA	
S20	M	94 (7.8)	11	83	Yes	Yes	No	HA	
S21	M	78 (6.5)	18	60	Yes	Yes	Yes	CI + HA	Nucleus CP 810
S22	F	91 (7.6)	26	65	Yes	No	No	HA	
S23	M	79 (6.6)	7	72	Yes	Yes	No	HA	
S24	F	123 (10.3)	6	117	Yes	Yes	Yes	CI + HA	Nucleus CP 810
S25	M	77 (6.4)	16	61	Yes	Yes	Yes	CI + HA	Nucleus freedom
S26	M	90 (7.5)	20	70	Yes	Yes	Yes	CI + HA	Nucleus CP 810
S27	F	116 (9.7)	19	97	Yes	Yes	No	HA	
S28	F	124 (10.3)	18	106	Yes	Yes	Yes	CI + HA	Nucleus CP 810

Mean		94.6 (7.9)	19.07	73.43					
SD		17.2 (1.4)	8.84	27.21					

*Duration of use of the device for HA + CI corresponds to the duration of utilization of CI*.

The music lessons consisted of the standard music courses delivered by the CEOP institute. They were performed by a music teacher and completed in small groups of five or six children. The training consisted of five progressive levels of difficulty, which increased after about 4 months of training at a given level. The first level focused on the binding between motor activities and auditory perception. Children used their voice and interacted with real instruments (drums, flutes, maracas, whistles, bells, keyboard, cymbales, and ocarinas) to discover what and how different sounds could be produced. The goal of this level was to explore the many sounds that could emanate from the voice or the various musical instruments by interacting with them in any way the children could imagine. This served to train invariant recognition of the instrument sounds. The second level consisted of the children engaging in sensorimotor activities. The children were encouraged to move their bodies along with the sounds they heard. They could, for example, sway to the rhythm of the music, shift from one foot to the other, rock on their chair. They were instructed to synchronize their movements to the rhythm of the music. Alternatively, a child performed a rhythmical movement with an instrument and their classmate had to adapt their musical activity to this movement. The third level consisted of exercises involving memory processes. For example, a child, hidden behind a folding screen, played a sequence of three or four notes with different instruments. The other children had to reproduce the same sequence by choosing the right instruments amongst several options. The fourth level consisted of analyzing the emotional value of musical pieces and the children’s feelings toward those pieces of music. The fifth level consisted of the children playing simple self-written pieces of music together. For example, a child chose a drum and decided to play it on the third and fourth beat of the measure while another child chose a bell and decided to play it on the second and fourth beat. The teacher sets the basic rhythm by playing each of the four beats of the measure on a drum for a period of time. The children played the music for two or three measures and repeated this play in a loop. When a child made a mistake, the others were invited to identify the problem.

### Procedure

**Figure 1:**
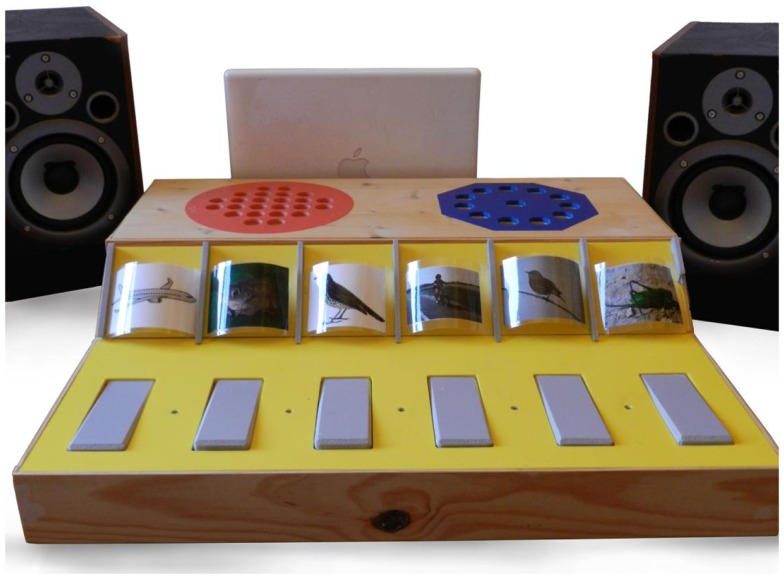
**Illustration of the “Sound in Hands” platform dressed for the identification task**.

The auditory performance of children was assessed with the “Sound in Hands” apparatus (see Figure 1; Rochette and Bigand, 2009), which is comprised of two speakers (70 cm apart and each 70 cm from the participant), a response platform, and a computer for sound generation. Sound level was adjusted to be comfortable for the children.

The Sound in Hands apparatus uses interactive games with a variety of environmental sounds to test four main operations involved in auditory perception and cognition (McAdams and Bigand, 1993): discrimination, identification, auditory scene analysis, and auditory working memory. All the tasks were carried out in a single session for a total duration of 30 min on average. Before each of the four tasks, children were invited to interact with the response platform used for the task, to familiarize themselves with its functioning (e.g., which sounds could be produced and how). The five tasks are further described below.

Identification and discrimination tasks evaluated the quality of the analysis of micro- and macro-temporal properties of relevant features of the signal. The discrimination task was done with the “magic hexagon” pierced by 12 holes (Figure 1). The children heard a continuous sound stream (i.e., the sound of a bulldozer). Five out of the 12 holes were magnetized. The introduction of a magnetic pawn (the “magic pawn”) in the magnetized holes modified the continuous auditory stream (to the sound of a maneuvering truck) whereas the introduction of the magic pawn in the not magnetized holes did not modify the auditory stream. A pile of blue and white pawns was at the child’s disposal. If a modification was perceived, the child had to fill the hole with a blue pawn. Conversely, if no modification was perceived, the child had to fill the hole with a white one. One point was given for each correct answer (maximum score = 12 points).

The identification task was executed with the keyboard. Each key produced an environmental sound, which was represented by a picture on the key (Figure 1). The child was presented with sounds in a randomized order and had to reproduce each sound (e.g., plane, frog, thrush, moped, nightingale, cicada) by pushing the corresponding key on the keyboard. Two points were given for a correct answer on the first attempt, and one point for a correct answer on the second attempt (maximum score = 12 points).

The auditory scene analysis task examined the blending and segregation of an auditory signal (Bregman, 1990). The auditory scene analysis task was conducted with a pegboard in which the 24 holes are filled with white magnetic pawns. Two auditory streams were simultaneously produced (cat and horse). Removing a pawn could shut down one of the two streams. When a change was detected, the child had to fill the hole with a yellow pawn. If the signal was not modified, the child filled the hole with a red pawn. One point was given for each correct answer (maximum score = 24 points).

In the auditory working memory task, the experimenter generated a sequence of two sounds that the child had to reproduce by ear in the same order, using the keyboard. Pictures on the keys indicated the sound that each key produced (steps in the water, crow, tit, rain, sheep, and goat). To avoid a contamination of the memory task by a process of identification of the sounds, children were authorized to proceed by trial and error to find which keys corresponded to the sounds before they provided the correct sequence to the experimenter. When the child succeeded for two trials in a row, an element was added to the sequence, up to a maximum of five elements. Two points were given for a correct reproduction of the sequence. Only one point was given if the child needed a second presentation of the sequence (maximum score = 16 points).

In the phonological discrimination task, children were presented with pairs of mono- or bi-syllabic nonsense words and they were asked to judge whether the two items of each pair were identical or different. The task was composed of three subtests. The first subtest was composed of pairs of mono-syllabic non-words, where the two non-words could vary in vowel composition (oral versus nasal, as in /o/ versus /Õ/; or “weak” vowels, as /i/ versus /y/) in the discordant pairs. The second subtest held vowels constant within pairs and assessed the discrimination of word-initial voiced (e.g., /b/) versus voiceless (e.g., /p/) consonants, again in mono-syllabic non-words. In the third subtest, discordant pairs were formed by placing voiced or voiceless consonants in the middle or at the end of bi-syllabic words. One point was given for each correct answer (maximum score = 36 points).

During the tasks, children were not given any feedback about their accuracy. The scores obtained in each task were converted into percentage of correct answers. To evaluate the effects of music lessons on our different measures, scores were analyzed with a Group (2) by Tasks (5) Repeated Measures ANOVA. For the tasks in which a training effect was found, multiple regressions were used to evaluate the effect of covariates such as the duration of music lessons, chronological age, the duration of deafness, the length of device use (length of auditory experience), perceptual threshold, and the type of device they use (cochlear implant versus hearing aids).

## Results

**Figure 2:**
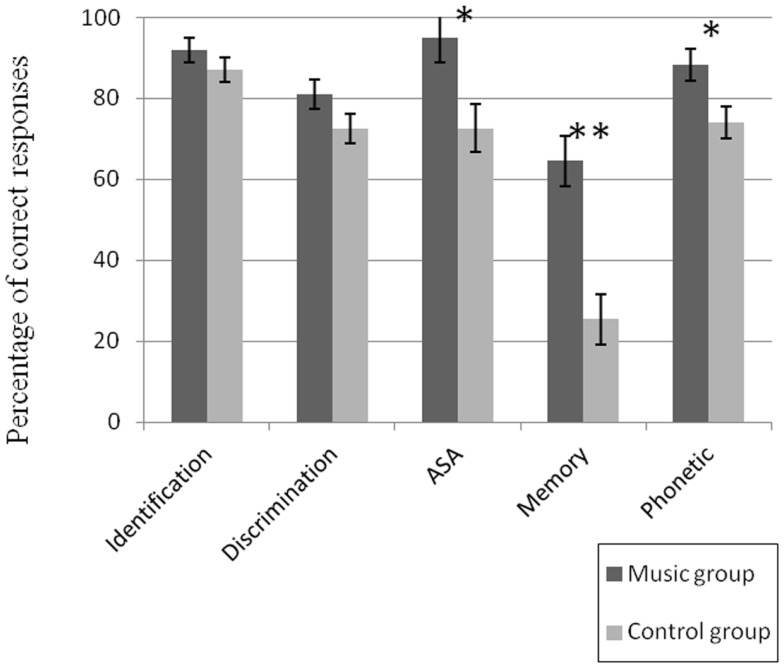
**Scores of auditory performance for the music and the control groups**. Stars represent significant differences between groups: *p < 0.05, **p < 0.01. Errors bars represent one standard error.

The results are presented in Figure 2. A main effect of Group [*F*(1, 26) = 14.55, *p* < 0.001, partial eta-squared η^2^ = 0.36] and an interaction between Group and Tasks [*F*(4, 104) = 43.12, *p* < 0.001] were found. Musically trained children obtained significantly higher scores in the auditory scene analysis task [*F*(1, 26) = 6.92, *p* < 0.05, η^2^ = 0.21], in the phonetic discrimination task [*F*(1, 26) = 6.74, *p* < 0.05, η^2^ = 0.21], and in the auditory working memory task [*F*(1, 26) = 19.79, *p* < 0.001, η^2^ = 0.43]. Groups did not significantly differ in discrimination and identification tasks, despite a tendency for higher scores in the music group.

In order to evaluate the respective contribution of various factors, which may impact the children’s task performance, multiple regressions were run for the tasks in which an effect of music training was found (i.e., auditory scene analysis, auditory working memory, and phonetic discrimination). The following factors, supposed to potentially influence task performance, were included in our regression model: music lessons (presence/absence), duration of music lessons (in months), chronological age (in months), duration of deafness (in months), length of device use (i.e., length of auditory experience; in months), perceptual threshold (yes/no), and type of device they use (cochlear implant/hearing aids). Table 2 presents the significant factors contributing to task performance. For auditory scene analysis, the results of the regression indicated that these factors explained 67% of the variance of the children’s scores. It was found that the presence of music lessons, the duration of music lessons, the age at correction (in favor of early corrected children), and the duration of use of the device (in favor of greater length of auditory experience) significantly predicted performance for auditory scene analysis. For the auditory working memory task, results of the regression showed that 60% of the variance was explained by our model. Performance for auditory working memory was significantly predicted by the presence of music lessons and the perceptual threshold at 4000 Hz (in favor of children that were able to hear 4000 Hz sounds at 50 dB). The percentage of variance explained for phonetic discrimination was only 27%, with the presence of music lessons as the only variable predicting scores.

**Table 2:** **Results from the multiple regressions including *R*^2^, *F*, β, and *t* statistics**.

Task	Music lessons (Yes/no)	Duration of music lessons (in months)	Age at correction (in months)	Duration of use of device (in months)	Threshold 4000 Hz (<50 dB)	Full Model
Auditory scene analysis	*t*(22) = 3.37,	*t*(22) = 2.69,	*t*(22) = 2.88,	*t*(22) = 5.11,		*R*^2^ = 0.67,
	β = 1.20**	β = −1.07*	β = 0.60**	β = 0.98***		*F*(5, 22) = 8.81***
Auditory working memory	*t*(23) = 5.16,				*t*(23) = 2.39,	*R*^2^ = 0.60,
	β = 0.83***				β = 0.47*	*F*(4, 23) = 8.57***
Phonological discrimination	*t*(24) = 2.63,					*R*^2^ = 0.27,
	β = 0.52*					*F*(3, 24) = 2.96,
						*p* = 0.053

*p < 0.05, **p < 0.01, ***p < 0.001.

Further correlations were performed to investigate the link between tasks using linguistic and non-linguistic sounds. Scores for phonetic discrimination were correlated with three of the tasks using environmental sounds: identification [*r*(26) = 0.42, *p* < 0.05], auditory scene analysis [*r*(26) = 0.47, *p* < 0.05], and auditory working memory [*r*(26) = 0.51, *p* < 0.01].

## Discussion

Despite advanced technology for the rehabilitation of hearing impairments, auditory training therapies remain crucial for children to learn to interpret auditory signals, create meaningful sound representation, and develop listening strategies. The goal of the present cross-sectional study was to assess the efficacy of music lessons to improve auditory perception and auditory cognition in deaf children. Four main processes of auditory cognition (discrimination, identification, auditory scene analysis, and auditory working memory) were evaluated using environmental sounds. Transfer to linguistic sounds was assessed using a phonetic discrimination task. Results showed that musically trained children performed significantly better than their non-musician counterparts for auditory scene analysis, auditory working memory, and phonetic discrimination. Moreover, multiple regressions revealed that music lessons was one of the main factors explaining children’s performance for the auditory scene analysis and the auditory working memory tasks (though other variables contributed to the performance in these tasks, such as age at correction, duration of use of device, and perceptual thresholds). Interestingly, for the phonetic discrimination task, only music lessons accounted for group differences, strongly suggesting the impact of musical training on transfer to the linguistic domain.

Although we observed a trend for better performance in musically trained children, identification and discrimination tasks using environmental sounds did not differ between groups. This could be due, in part, to the fact that low-level of auditory perception is already intensively trained in deaf children through their regular therapies. For example, early stages of typical speech therapy require children to examine sounds parameters such as changes in frequency, tempo, duration, or timbre in stimuli that often include environmental sounds. This might explain the ceiling effect we observed in the identification task and might have hidden potential differences between groups in our two perceptive tasks using environmental sounds. However, when examining auditory perceptual abilities using linguistic sounds (phonological discrimination), we observed better performance for musically trained children compared to controls. This suggests that music training contributes to develop abilities at a perceptual level and allows children to create more efficient auditory representations of sounds. Moreover, even when including other factors that may contribute to performance on the linguistic task, only musical training significantly influenced performance. Rhythm exercises from the music curriculum may have contributed to this effect. Rhythm has been shown to be a crucial gateway for phonological representations (Leong et al., 2011; Rauscher and Hinton, 2011) and strong associations between poor perception of musical meter, reading, and phonological representations were found in dyslexic children (Huss et al., 2011). In a recent study using the child version of the Montreal Battery of Evaluation of Amusia (MBEA; Peretz et al., 2003), Hopyan et al. (2012) found that pediatric cochlear implant users performed significantly below the matched normal-hearing children in the rhythm subtest. The rhythm component of the music lessons may have contributed to the development of the deaf children’s sensitivity to the rhythmic structure in speech which, in turn, may have allowed for the development of higher quality of phonological representations (Kotz and Schwartze, 2010).

Better performance in the musically trained children in the auditory scene analysis task suggests that higher levels of auditory perception such as stream segregation and the representation of auditory scenes are enhanced by music training. These results may be due to the acoustic richness of musical training sessions and the graded nature of the training (i.e., increased task difficulty at each stage). For example, in the exercises in which children created musical pieces, children developed their ability to analyze auditory streams composed of at least two sources. This may have contributed to enhanced listening strategies. The current finding is consistent with previous studies showing that abilities related to auditory scene analysis, such as speech in noise perception, which is impaired in deaf children (Kral and Sharma, 2012), can be enhanced with auditory training therapies (Strait and Kraus, 2014). Interestingly, in the normal-hearing adults, Skoe and Kraus (2012) showed that a limited period of music practice in childhood (3 years on average) influences how the brain further encodes and processes sounds later in life. Thus, in deaf children, it is possible that listening strategies taught at a young age would have a life-long impact, if the children keep using these strategies beyond the context of musical lessons.

There are two potential interpretations for the results from the auditory working memory performance, in which musically trained children show better performance than controls. First, enhanced auditory representation and listening strategies, as evident by better performance in phonological discrimination and auditory scene analysis by the musically trained children, may have facilitated encoding and storage in working memory. Some studies highlighted a causal link between poor encoding of sounds and difficulties in working memory in deaf children (Pisoni and Cleary, 2003; Pisoni et al., 2011; Nittrouer et al., 2013). This is consistent with the observation of enhanced auditory (but not necessarily visual) working memory in normal-hearing children and adults after musical practice, compared to matched non-musician, (in children: Moreno et al., 2011; Strait et al., 2012; in adults: Berti et al., 2006; Hansen et al., 2013; Parbery-Clark et al., 2009). Alternatively, the music exercises relied on working memory for an important part and this might have enhanced general working memory ability, beyond auditory-specific working memory (see George and Coch, 2011 for enhanced visuo-spatial memory in normal hearing adult musicians). This would be consistent with studies showing that music training improves various high level cognitive processes in normal-hearing children (e.g., Schellenberg, 2004). Future studies could test whether or not musical training could generally improve working memory processes of deaf children in a non-auditory context.

Surprisingly, we did not found any effect of type of device or amodal versus bimodal aid (as CI users also wore HA in the contralateral ear). Due to limited spectral resolution and reduced temporal fine-grained structure in cochlear implants (Kong et al., 2005; Zeng et al., 2008), encoding pitch information remains a challenge (McDermott, 2004; Looi et al., 2008) and a hearing aid in the non-implanted ear could have positively influenced music processing and thus the gain from music training (Kong et al., 2005). Further studies will explore interactions between music training and type of device to determine which profiles of children would benefit most from the training.

The main limitation of the study is the cross-sectional nature of the study design, which does not allow to draw causal conclusions regarding musical training as the present results could be due, at least in part, to pre-existing differences between groups or confounding factors (e.g., perceptive threshold levels, chronological age). Although our multiple regression analysis suggested music factor as an influential factor even after accounting for other possible confounds, it is possible that other factors not examined presently could underlie the group differences reported here. In addition, schooling programs would differ between the two groups for 50% of their time, which could also have influenced the children’s performance. Longitudinal studies using a randomized controlled trial design and blind testers are necessary to further replicate these findings. Further study would also need to investigate effects of training at the neural level. Using a deviant detection task (tone, duration, intensity, and timbre) in pediatric cochlear implant users that were not musically trained, Torppa et al. (2012) observed smaller amplitudes for the P1, MMN, and P3a components as well as longer latency of the MMN than in matched normal hearing children. Further studies will investigate if music training could improve these neural indices in deaf children.

As a final note, it is important to mention that a great advantage of music is to be an enjoyable stimulus. Contrary to the post-lingually cochlear-implanted adults who report little appreciation in listening to music due to the spectral limitation of the device and the comparison with their prior musical knowledge (McDermott, 2004; Looi et al., 2008), deaf children show interest and pleasure in listening to music (Nakata et al., 2005). They appreciate activities involving music such as listening to music, dancing, singing, or instrumental practice (Gfeller et al., 1999). As in normal-hearing children, music has the power to modify their mood (Hopyan-Misakyan et al., 2009). Moreover, motivation and enjoyment have been shown to improve learning effects and to boost brain plasticity (Sutoo and Akiyama, 2004). Thus, while clinical rehabilitation with children remains a challenge due to lack of engaging training program and difficulty to keep children’s attention and motivation, music could represent a highly effective rehabilitation tool (Bruner, 1960; Kim, 2013).

To conclude, this study provides evidence that music may constitute a relevant tool for deaf children rehabilitation. Music lessons promote improved auditory perception and the development of finer sound representations and listening strategies. Moreover, improvements in these processes appear to have down-stream effects on higher-order auditory cognition (i.e., auditory working memory). Interestingly, these results provide support for cross-domain transfer into the linguistic domain. Therefore, musical training may be an interesting and useful vehicle for enhancing the basic linguistic processes that are necessary for improving higher-order linguistic processes (i.e., vocabulary and reading abilities).

## Conflict of Interest Statement

The authors declare that the research was conducted in the absence of any commercial or financial relationships that could be construed as a potential conflict of interest.

## Footnotes

^1^French criteria for pediatric cochlear implantation in pre-lingually deaf children are: profound or total deafness (loss of >91 dB), perceptive thresholds at 2000 and 4000 Hz >60 dB after 3–6 months of regular bearing of devices, no pathologies of middle and external ears, no morphological disease of the cochlea (as measured by a complete radiological assessment), and no neurological disease (as measured by magnetic resonance imaging).

## References

[B1] Anvari S. H. Trainor L. J. Woodside J. Levy B. A. (2002). Relations among musical skills, phonological processing, and early reading ability in preschool children. J. Exp. Child Psychol. 83, 111–130 10.1016/S0022-0965(02)00124-8 12408958

[B2] Berti S. Münzer S. Schröger E. Pechmann T. (2006). Different interference effects in musicians and a control group. Exp. Psychol. 53, 111–116 10.1027/1618-3169.53.2.111 16909935

[B3] Besson M. Chobert J. Marie C. (2011). Transfer of training between music and speech: common processing, attention and memory. Front. Psychol. 2:94 10.3389/fpsyg.2011.00094 21738519 PMC3125524

[B4] Bilhartz T. D. Bruhn R. A. Olson J. E. (1999). The effect of early music training on child cognitive development. J. Appl. Dev. Psychol. 20, 615–636 10.1016/S0193-3973(99)00033-7

[B5] Bregman A. S. (1990). Auditory Scene Analysis: The Perceptual Organization of Sound. Cambridge, MA: MIT Press

[B6] Bruner J. (ed.) (1960). The Process of Education. Cambridge, MA: Harvard University Press

[B7] Chen J. K.-C. Chuang A. Y. C. McMahon C. Hsieh T.-H. Li L. P.-H. (2010). Music training improves pitch perception in prelingually deafened children with cochlear implants. Pediatrics 125, 793–800 10.1542/peds.2008-3620 20211951

[B8] Chen S. H. Liu H. Xu Y. Larson C. R. (2007). Voice F0 responses to pitch-shifted voice feedback during English speech. J. Acoust. Soc. Am. 121, 1157–1163 10.1121/1.2404624 17348536

[B9] Chobert J. François C. Velay J.-L. Besson M. (2012). Twelve months of active musical training in 8- to 10-year-old children enhances the preattentive processing of syllabic duration and voice onset time. Cereb. Cortex 24, 956–967 10.1093/cercor/bhs377 23236208

[B10] Degé F. Schwarzer G. (2011). The effect of a music program on phonological awareness in preschoolers. Front. Psychol. 2:124 10.3389/fpsyg.2011.00124 21734895 PMC3121007

[B11] Geers A. E. Nicholas J. G. Moog J. S. (2007). Estimating the influence of cochlear implantation on language development in children. Audiol. Med. 5, 262–273 10.1080/16513860701659404 21243079 PMC3020793

[B12] George E. M. Coch D. (2011). Music training and working memory: an ERP study. Neuropsychologia 49, 1083–1094 10.1016/j.neuropsychologia.2011.02.001 21315092

[B13] Gfeller K. Witt S. Spencer L. Stordhal J. Tomblin B. (1999). Musical involvement and enjoyment of children who use cochlear implants. Volta Rev. 100, 213–233

[B14] Hansen M. Wallentin M. Vuust P. (2013). Working memory and musical competence of musicians and non-musicians. Psychol. Music 41, 779–793 10.1177/0305735612452186

[B15] Havy M. Nazzi T. Bertoncini J. (2013). Phonetic processing during the acquisition of new words in 3-to-6 year-old-French-speaking deaf children with cochlear implants. J. Commun. Disord. 46, 181–192 10.1016/j.jcomdis.2012.12.002 23295076

[B16] Hopyan T. Peretz I. Chan L. P. Papsin B. C. Gordon K. A. (2012). Children using cochlear implants capitalize on acoustical hearing for music perception. Front. Psychol. 3:425 10.3389/fpsyg.2012.00425 23133430 PMC3490327

[B17] Hopyan-Misakyan T. M. Gordon K. A. Dennis M. Papsin B. C. (2009). Recognition of affective speech prosody and facial affect in deaf children with unilateral right cochlear implants. Child Neuropsychol. 15, 136–146 10.1080/09297040802403682 18828045

[B18] Huss M. Verney J. P. Fosker T. Mead N. Goswami U. (2011). Music, rhythm, rise time perception and developmental dyslexia: perception of musical meter predicts reading and phonology. Cortex 47, 674–689 10.1016/j.cortex.2010.07.010 20843509

[B19] Hyde K. L. Lerch J. Norton A. Forgeard M. Winner E. Evans A. C. (2009). Musical training shapes structural brain development. J. Neurosci. 29, 3019–3025 10.1523/JNEUROSCI.5118-08.2009 19279238 PMC2996392

[B20] Kim S.-I. (2013). Neuroscientific model of motivational process. Front. Psychol. 4:98 10.3389/fpsyg.2013.00098 23459598 PMC3586760

[B21] Koelsch S. Schulze K. Sammler D. Fritz T. Müller K. Gruber O. (2009). Functional architecture of verbal and tonal working memory: an FMRI study. Hum. Brain Mapp. 30, 859–873 10.1002/hbm.20550 18330870 PMC6871123

[B22] Kong Y. Y. Stickney G. S. Zeng F. G. (2005). Speech and melody recognition in binaurally combined acoustic and electric hearing. J. Acoust. Soc. Am. 117, 1351–1361 10.1121/1.1857526 15807023

[B23] Kotz S. A. Schwartze M. (2010). Cortical speech processing unplugged: a timely subcortico-cortical framework. Trends Cogn. Sci. 14, 392–399 10.1016/j.tics.2010.06.005 20655802

[B24] Kral A. (2013). Auditory critical periods: a review from system’s perspective. Neuroscience 247, 117–133 10.1016/j.neuroscience.2013.05.021 23707979

[B25] Kral A. Eggermont J. J. (2007). What’s to lose and what’s to learn: development under auditory deprivation, cochlear implants and limits of cortical plasticity. Brain Res. Rev. 56, 259–269 10.1016/j.brainresrev.2007.07.021 17950463

[B26] Kral A. Hartmann R. Tillein J. Heide S. (2000). Congenital auditory deprivation reduces synaptic activity within the auditory cortex in a layer-specific manner. Cereb. Cortex 10, 714–726 10.1093/cercor/10.7.714 10906318

[B27] Kral A. Sharma A. (2012). Developmental neuroplasticity after cochlear implantation. Trends Neurosci. 35, 111–122 10.1016/j.tins.2011.09.004 22104561 PMC3561718

[B28] Kühnis J. Elmer S. Meyer M. Jäncke L. (2013). The encoding of vowels and temporal speech cues in the auditory cortex of professional musicians: an EEG study. Neuropsychologia 51, 1608–1618 10.1016/j.neuropsychologia.2013.04.007 23664833

[B29] Leong V. Hämäläinen J. Soltész F. Goswami U. (2011). Rise time perception and detection of syllable stress in adults with developmental dyslexia. J. Mem. Lang. 64, 59–73 10.1016/j.jml.2010.09.003

[B30] Looi V. McDermott H. McKay C. (2008). Music perception of cochlear implant users compared with that of hearing aids users. Ear Hear. 29, 421–434 10.1097/AUD.0b013e31816a0d0b 18344870

[B31] McAdams S. Bigand E. (1993). Thinking in Sound: The Cognitive Psychology of Human Audition. Oxford: Oxford University Press

[B32] McDermott H. J. (2004). Music perception with cochlear implants: a review. Trends Amplif. 8, 49–82 10.1177/108471380400800203 15497033 PMC4111359

[B33] Moreno S. (2009). Can music influence language and cognition? Contemp. Music Rev. 28, 329–345 10.1080/07494460903404410

[B34] Moreno S. Bialystok E. Barac R. Schellenberg E. G. Cepeda N. Chau T. (2011). Short-term music training enhances verbal intelligence and executive function. Psychol. Sci. 22, 1425–1433 10.1177/0956797611416999 21969312 PMC3449320

[B35] Moreno S. Marques C. Santos A. Santos M. Castro S. L. Besson M. (2009). Musical training influences linguistic abilities in 8-year-old children: more evidence for brain plasticity. Cereb. Cortex 19, 712–723 10.1093/cercor/bhn120 18832336

[B36] Nakata T. Trehub S. Kanda Y. Shibasaki A. Schellenberg E. G. (2005). Music recognition by Japanese children with cochlear implants. J. Physiol. Anthropol. Appl. Human Sci. 24, 29–32 10.2114/jpa.24.29 15684539

[B37] Nittrouer S. Caldwell-Tarr A. Lowenstein J. H. (2013). Working memory in children with cochlear implants: problems are in storage, not processing. Int. J. Pediatr. Otorhinolaryngol. 77, 1886–1898 10.1016/j.ijporl.2013.09.001 24090697 PMC3855408

[B38] Nutley S. B. Darki F. Klingberg T. (2014). Music practice is associated with development of working memory during childhood and adolescence. Front. Hum. Neurosci. 7:926 10.3389/fnhum.2013.00926 24431997 PMC3882720

[B39] Parbery-Clark A. Skoe E. Lam C. Kraus N. (2009). Musician enhancement for speech-in-noise. Ear Hear. 30, 653–661 10.1097/AUD.0b013e3181b412e9 19734788

[B40] Patel A. D. (2008). Music, Language and the Brain. New York, NY: Oxford University Press

[B41] Peretz I. Champod A.-S. Hyde K. L. (2003). Varieties of musical disorders the montreal battery of evaluation of amusia. Ann. N. Y. Acad. Sci. 999, 58–75 10.1196/annals.1284.006 14681118

[B42] Petersen B. Mortensen M. V. Hansen M. Vuust P. (2012). Singing in the key of life – a pilot study on effects of musical ear training after cochlear implantation. Psychomusicology 22, 134–151 10.1111/j.1749-6632.2009.04796.x 19673820

[B43] Peterson N. R. Pisoni D. B. Miyamotoa R. T. (2010). Cochlear implants and spoken language processing abilities: review and assessment of the literature. Restor. Neurol. Neurosci. 28, 237–250 10.3233/RNN-2010-0535 20404411 PMC2947146

[B44] Pisoni D. B. Cleary M. (2003). Measures of working memory span and verbal rehearsal speed in deaf children after cochlear implantation. Ear Hear. 24(1 Suppl.), 106S–120S 10.1097/01.AUD.0000051692.05140.8E 12612485 PMC3434463

[B45] Pisoni D. B. Conway C. M. Kronenberger W. G. Horn D. L. Karpicke J. Henning S. C. (2008). “Efficacy and effectiveness of cochlear implants in deaf children,” in Deaf Cognition: Foundations and Outcomes, eds Marschark M. Hauser P. (New York, NY: Oxford University Press), 52–101

[B46] Pisoni D. B. Kronenberger W. G. Roman A. S. Geers A. E. (2011). Measures of digit span and verbal rehearsal speed in deaf children after more than 10 years of cochlear implantation. Ear Hear. 32(1 Suppl.), 60S–74S 10.1097/AUD.0b013e3181ffd58e 21832890 PMC3080130

[B47] Rauscher F. H. Hinton S. C. (2011). Music instruction and its diverse extra-musical benefits. Music Percept. 29, 215–226 10.1525/mp.2011.29.2.215

[B48] Rochette F. Bigand E. (2009). Long-term effects of auditory training in severely or profoundly deaf children. Ann. N. Y. Acad. Sci. 1169, 195–198 10.1111/j.1749-6632.2009.04793.x 19673780

[B49] Schellenberg E. G. (2004). Music lessons enhance IQ. Psychol. Sci. 15, 511–514 10.1111/j.0956-7976.2004.00711.x 15270994

[B50] Sharma A. Dorman M. F. Spahr A. J. (2002). A sensitive period for the development of the central auditory system in children with cochlear implants; implications for age of implantation. Ear Hear. 23, 532–539 10.1097/00003446-200212000-00004 12476090

[B51] Skoe E. Kraus N. (2012). A little goes a long way: how the adult brain is shaped by musical training in childhood. J. Neurosci. 32, 11507–11510 10.1523/JNEUROSCI.1949-12.2012 22915097 PMC6703757

[B52] Strait D. Kraus N. (2014). Biological impact of auditory expertise across the life span: musicians as a model of auditory learning. Hear. Res. 308, 109–121 10.1016/j.heares.2013.08.004 23988583 PMC3947192

[B53] Strait D. L. Parbery-Clark A. Hittner E. Kraus N. (2012). Musical training during early childhood enhances the neural encoding of speech in noise. Brain Lang. 123, 191–201 10.1016/j.bandl.2012.09.001 23102977 PMC3502676

[B54] Sutoo D. Akiyama K. (2004). Music improves dopaminergic neurotransmission: demonstration based on the effect of music on blood pressure regulation. Brain Res. 1016, 255–262 10.1016/j.brainres.2004.05.018 15246862

[B55] Tillmann B. Koelsch S. Escoffier N. Bigand E. Lalitte P. Friederici A. D. (2006). Cognitive priming in sung and instrumental music: activation of inferior frontal cortex. Neuroimage 31, 1771–1782 10.1016/j.neuroimage.2006.02.028 16624581

[B56] Torppa R. Faulkner A. Huotilainen M. Järvikivi J. Lipsanen J. Laasonen M. (2014). The perception of prosody and associated auditory cues in early-implanted children: the role of auditory working memory and musical activities. Int. J. Audiol. 53, 182–191 10.3109/14992027.2013.872302 24460045

[B57] Torppa R. Salo E. Makkonen T. Loimo H. Pykäläinen J. Lipsanen J. (2012). Cortical processing of musical sounds in children with cochlear implants. Neurophysiol. Clin. 123, 1966–1979 10.1016/j.clinph.2012.03.008 22554786

[B58] Trainor L. J. Shahin A. J. Roberts L. E. (2003). Effects of musical training on auditory cortex in children. Ann. N. Y. Acad. Sci. 999, 506–513 10.1196/annals.1284.061 14681174

[B59] Wan C. Y. Schlaug G. (2010). Music making as a tool for promoting brain plasticity across the life span. Neuroscientist 16, 566–577 10.1177/1073858410377805 20889966 PMC2996135

[B60] Wu J. L. Yang H. M. Lin Y. H. Fu Q. J. (2007). Effects of computer-assisted speech training on Mandarin-speaking hearing-impaired children. Audiol. Neurootol. 12, 307–312 10.1159/000103211 17536199 PMC3580209

[B61] Yucel E. Sennaroglu G. Belgin E. (2009). The family oriented musical training for children with cochlear implants: speech and musical perception results of two year follow-up. Int. J. Pediatr. Otorhinolaryngol. 73, 1043–1052 10.1016/j.ijporl.2009.04.009 19411117

[B62] Zeng F. G. Rebscher S. Harrison W. V. Sun X. Feng H. (2008). Cochlear implants: system design, integration and evaluation. IEEE Rev. Biomed. Eng. 1, 115–142 10.1109/RBME.2008.2008250 19946565 PMC2782849

